# Cardiovascular Complications of Down Syndrome: Scoping Review and Expert Consensus

**DOI:** 10.1161/CIRCULATIONAHA.122.059706

**Published:** 2023-01-31

**Authors:** Konstantinos Dimopoulos, Andrew Constantine, Paul Clift, Robin Condliffe, Shahin Moledina, Katrijn Jansen, Ryo Inuzuka, Gruschen R. Veldtman, Clifford L. Cua, Edgar Lik Wui Tay, Alexander R. Opotowsky, George Giannakoulas, Rafael Alonso-Gonzalez, Rachael Cordina, George Capone, Judith Namuyonga, Charmaine H. Scott, Michele D’Alto, Francisco J. Gamero, Brian Chicoine, Hong Gu, Alisa Limsuwan, Tosin Majekodunmi, Werner Budts, Gerry Coghlan, Craig S. Broberg

**Affiliations:** Adult Congenital Heart Centre and Centre for Pulmonary Hypertension, Royal Brompton Hospital, Royal Brompton and Harefield Hospitals, Guy’s and St Thomas’ NHS Foundation Trust, London, United Kingdom (K.D., A.C.).; National Heart and Lung Institute, Imperial College London, United Kingdom (K.D., A.C.).; Department of Cardiology, Queen Elizabeth Hospital Birmingham, United Kingdom (P.C.).; Pulmonary Vascular Disease Unit, Royal Hallamshire Hospital, Sheffield, United Kingdom (R.C.).; National Paediatric Pulmonary Hypertension Service UK, Great Ormond Street Hospital for Children NHS Foundation Trust, London, United Kingdom (S.M.).; Institute of Cardiovascular Science, University College London, United Kingdom (S.M.).; Adult Congenital and Paediatric Heart Unit, Freeman Hospital Newcastle Upon Tyne Hospitals NHS Foundation Trust, Newcastle upon Tyne, United Kingdom (K.J.).; Population Health Sciences Institute, Newcastle University, Newcastle upon Tyne, United Kingdom (K.J.).; Department of Pediatrics, The University of Tokyo Hospital, Japan (R.I.).; Scottish Adult Congenital Cardiac Service, Golden Jubilee Hospital, Glasgow, Scotland, United Kingdom (G.R.V.).; The Heart Center, Nationwide Children’s Hospital, Columbus, OH (C.L.C.).; Department of Cardiology, National University Hospital Singapore (E.T.L.W.).; The Heart Institute, Department of Pediatrics, Cincinnati Children’s Hospital, University of Cincinnati College of Medicine, OH (A.R.O.).; Department of Cardiology, AHEPA University Hospital School of Medicine, Aristotle University of Thessaloniki, Greece (G.G.).; Division of Cardiology, Toronto General Hospital, University Health Network, Peter Munk Cardiovascular Center, University of Toronto, Canada (R.A.-G.).; Toronto Adult Congenital Heart Disease Program, Canada (R.A.-G.).; Department of Cardiology, Royal Prince Alfred Hospital and Sydney Medical School, University of Sydney, New South Wales, Australia (R.C.).; Down Syndrome Clinical and Research Center, Kennedy Krieger Institute, Baltimore, MD (G. Capone).; Johns Hopkins School of Medicine, Baltimore, MD (G. Capone).; Department of Paediatric Cardiology, Uganda Heart Institute, Kampala (J.N.).; Department of Paediatrics and Child Health, Makerere University College of Health Sciences, Kampala, Uganda (J.N.).; University Hospital of the West Indies, Kingston, Jamaica (C.H.S.).; Department of Cardiology, University “L. Vanvitelli”–Monaldi Hospital, Naples, Italy (M.D.).; Department of Cardiovascular Surgery, Benjamin Bloom Children’s Hospital, El Salvador (F.J.G.).; Advocate Medical Group Adult Down Syndrome Center, Park Ridge, IL (B.C.).; Department of Pediatric Cardiology, Beijing Anzhen Hospital, Capital Medical University, China (H.G.).; Division of Pediatric Cardiology, Department of Pediatrics, Ramathibodi Hospital, Mahidol University, Bangkok, Thailand (A.L.).; Department of Cardiology, Euracare Multi-specialist Hospital, Nigeria (T.M.).; Division of Congenital and Structural Cardiology, University Hospitals Leuven, and Department of Cardiovascular Science, Catholic University Leuven, Belgium (W.B.).; Department of Cardiology, Royal Free Hospital, London, United Kingdom (G. Coghlan).; Knight Cardiovascular Institute, Oregon Health and Science University, Portland (C.S.B.).; Adult Congenital Heart Centre and Centre for Pulmonary Hypertension, Royal Brompton and Harefield Hospitals, and National Heart and Lung Institute; Adult Congenital Heart Centre and Centre for Pulmonary Hypertension, Royal Brompton and Harefield Hospitals, and National Heart and Lung Institute, Imperial College London; Department of Cardiology, Queen Elizabeth Hospital Birmingham; Pulmonary Vascular Disease Unit, Royal Hallamshire Hospital; National Paediatric Pulmonary Hypertension Service UK, Great Ormond Street Hospital for Children NHS Foundation Trust and Institute of Cardiovascular Science, University College London; Adult Congenital and Paediatric Heart Unit, Freeman Hospital Newcastle Upon Tyne Hospitals NHS Foundation Trust and Population Health Sciences Institute, Newcastle University, Newcastle upon Tyne, United Kingdom

**Keywords:** cardiovascular diseases, Down syndrome, heart defects, congenital, hypertension, pulmonary

## Abstract

Cardiovascular disease is a leading cause of morbidity and mortality in individuals with Down syndrome. Congenital heart disease is the most common cardiovascular condition in this group, present in up to 50% of people with Down syndrome and contributing to poor outcomes. Additional factors contributing to cardiovascular outcomes include pulmonary hypertension; coexistent pulmonary, endocrine, and metabolic diseases; and risk factors for atherosclerotic disease. Moreover, disparities in the cardiovascular care of people with Down syndrome compared with the general population, which vary across different geographies and health care systems, further contribute to cardiovascular mortality; this issue is often overlooked by the wider medical community. This review focuses on the diagnosis, prevalence, and management of cardiovascular disease encountered in people with Down syndrome and summarizes available evidence in 10 key areas relating to Down syndrome and cardiac disease, from prenatal diagnosis to disparities in care in areas of differing resource availability. All specialists and nonspecialist clinicians providing care for people with Down syndrome should be aware of best clinical practice in all aspects of care of this distinct population.

Down syndrome (DS) is the most common chromosomal abnormality, present in 16 per 10 000 live births.^1^ Cardiovascular disease is common in people with DS and includes various types of congenital heart disease (CHD), a predisposition to the development of pulmonary hypertension (PH), and DS-related comorbidity, such as obesity and sleep apnea, which can affect the cardiovascular system. Even though cardiovascular conditions associated with DS are well-described, detailed DS-specific expert opinion on clinical recognition, diagnosis, and management of cardiovascular disease are lacking.

We present a scoping review of the literature, focusing on recent advances and modern clinical practices in the management of cardiovascular disorders encountered in people with DS, with clinical expert opinion on the basis of the best available information relevant to high-, middle-, and low-income countries.

## Methods

A scoping review of all published reports relating to cardiovascular disease in DS was performed in accordance with the PRISMA (Preferred Reporting Items for Systematic Reviews and Meta-Analyses) guidelines.^2^ A panel of experts in cardiac disease and DS developed a list of queries for the scoping review and expert discussion on various aspects of heart disease in DS:

1. What is the incidence of CHD? Are there changes in incidence over time and place? Which forms of CHD are most common?
2. What is the best practice for prenatal and neonatal diagnosis and what management needs arise during this period?
3. What is the optimal timing of repair of CHD (for each of the common conditions) and the risk of developing PH?
4. What are the perioperative risks, complications, and optimal care of CHD repair?
5. What are the sequelae of CHD, including residual lesions, PH, heart failure, and the need for reintervention? What other mechanisms can cause or contribute to the development of PH beyond CHD? What are the long-term outcomes?
6. What is the optimal follow-up and long-term care for adolescents and adults with CHD or PH?
7. What is the influence of acquired heart disease and noncardiac comorbidities on management and decision making related to CHD?
8. What is the influence of learning disabilities on the practical management of individuals with CHD?
9. What is the optimal approach to diagnose and manage cardiac disease in areas of different resource availability, including health care resources?
10. What are the unmet needs and challenges of research?

PubMed, Web of Science, and the Cochrane library were searched for articles relevant to these topics (Expanded Online Appendix for Additional Methodologic Details in the Supplemental Material, Tables S1 through S6, and Figure S1) and identified 1662 articles. Two experienced independent clinicians (K.D. and A.C.) used the Covidence platform to screen and select 460 relevant articles, which were then grouped according to their relevance to each of the research questions and made available to the working group.

## Results

Expert comments and statements of good practice in the 10 key areas presented in the following are summarized in Table 1.

**Table 1.**
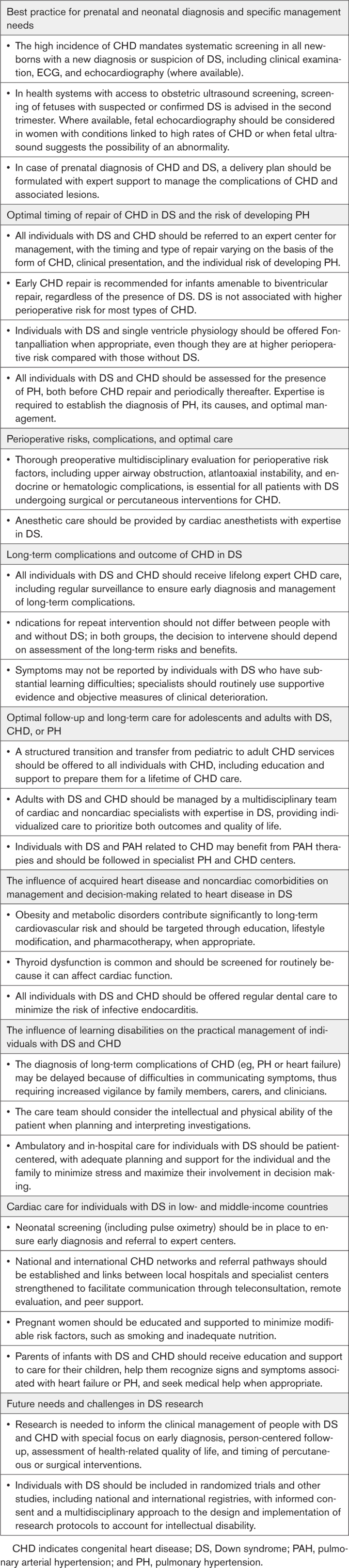
Expert Comments and Statements of Good Practice in Key Areas Relating to DS and Cardiovascular Disease

### Incidence and Types of CHD in DS

The presence of DS is associated with a 40 to 50 times greater likelihood of CHD than in the general population.^3,4^ Although this association was first documented in 1894,^5^ it was not until the 1950s that clinical studies more precisely defined the types of lesions, their prevalence, prognosis, and treatment implications. These initial Baltimore–Washington and New South Wales studies documented that atrioventricular septal defects (AVSDs) and ventricular septal defects (VSDs) formed 76% of the CHD seen in DS.^6,7^ Approximately half of live-born infants with DS are diagnosed with CHD, compared with ~1% in the general population, but the precise incidence of CHD in DS is unclear. Even in population-based studies that minimize referral bias, the reported incidence of CHD in DS varies widely with time and place, from 23% to 79% (Table S7).^4,8–26^ In studies using diagnostic ultrasound, CHD is seen in 29% to 56% of karyotype-proven DS cases.^3,13,27^

Variation in the incidence among studies is partly attributed to underascertainment of CHD in the neonatal and pediatric surveillance system, improved detection of CHD with advances in ultrasound technology, and inclusion in studies of minor CHD lesions, such as closing or small patent ductus arteriosus (PDA).^14,15,19^ Moreover, many environmental factors are known to increase the risk of CHD in DS, such as maternal smoking, obesity, and lack of folic acid supplementation in pregnancy.^26,28^ Therefore, the incidence of CHD in DS in a population-based study is affected by the maternal characteristics of the study population. The incidence of CHD in DS appears to have remained stable over time.^12,13,16,24,26^

AVSD was the most common form of CHD in 11 out of 15 studies shown in Table S7. Other frequently encountered lesions include isolated tetralogy of Fallot (ToF) in ~13%, combined AVSD and ToF in ~9% of cases, and isolated VSD in 4% to 17%.^3,27^ Bergström and colleagues^26^ reported a change in the distribution of CHD in DS over time, with a shift toward more simple lesions in recent years. This could be a bias toward better survival in simple lesions, but could also reflect a higher rate of prenatal diagnosis and a greater likelihood of termination of pregnancy for more complicated defects.

### Best Practice for Prenatal and Neonatal Diagnosis and Specific Management Needs

#### Antenatal Diagnosis and Testing

The advent of diagnostic ultrasound techniques, together with the refinement of its 2- and 3-dimensional and Doppler capabilities, has enabled widespread application to the diagnosis of DS and associated CHD, informing antenatal and postnatal counseling and care in developed countries. Where available, routine antenatal ultrasound screening is recommended in the second trimester (at 18 to 22 weeks) using a modern screening protocol that incorporates views of the heart.^29^ In expert hands, detailed fetal ultrasound scans can have an excellent detection rate for CHD, limiting the need for fetal echocardiography.^30^ Detection rates during routine fetal ultrasound may, however, vary depending on the type of defect, experience of the sonographer, and the screening protocol. —US-based studies have shown that, even though the large majority of mothers of children with CHD had undergone a second or third trimester antenatal ultrasound, fewer than a third had received a prenatal diagnosis of CHD.^31,32^

In health care systems with access, a detailed fetal echocardiogram may be indicated when a fetal ultrasound suggests the possibility of a cardiovascular abnormality or in the presence of conditions such as maternal diabetes (diagnosed before pregnancy or during the first trimester), uncontrolled phenylketonuria, first trimester rubella infection, fetal karyotype abnormality, fetal hydrops or effusions, or factors including maternal medication (eg, angiotensin-converting enzyme inhibitors, retinoids), or a strong family history of CHD.^13,29^ In experienced hands, fetal echocardiography can accurately identify most complex types of CHD in >90% of fetuses.^33^ In 1 recent fetal echocardiography-based study, a normal scan provided a negative predictive value of 100% for a diagnosis of complex CHD. When compared with neonatal echocardiography, however, second trimester fetal echocardiography may fail to identify smaller atrial septal defects (ASDs) or VSDs, and may underestimate the degree of aortic arch or ventricular hypoplasia, especially in the absence of serial antenatal scans.^33^

Prenatal genetic testing has used conventional metaphase chromosome banding of fetal cells obtained by amniocentesis or chorionic villus sampling. In recent years, fluorescence in situ hybridization has emerged as the preferred technique for the detection of chromosomal abnormalities. Nowadays, noninvasive prenatal testing has largely replaced those techniques, using a combination of fetal ultrasound for nuchal translucency (to detect increased thickness of the fluid-filled subcutaneous space located at the back of the fetal neck in the late first and early second trimesters, related to numerous fetal abnormalities), maternal blood testing, and cell-free DNA, which has a detection rate of 99.5% for trisomy 21.^34^ Invasive testing with amniocentesis or chorionic villus sampling is reserved for confirmatory testing in a minority of patients considered at high risk after undergoing noninvasive testing. Timely identification of DS and associated CHD may influence the decision to proceed with the pregnancy. Information from fetal imaging informs the counseling session in which the option of termination of pregnancy is often discussed.

Practices vary among countries and many of the resources mentioned, including fetal imaging and other prenatal diagnostic testing, may not be available in developing countries. Moreover, there is a cost burden attached to fetal screening, which varies by country and health care system. In settings where fetal screening is not widely accessible, neonatal screening for signs of DS or CHD becomes essential.

#### Postnatal Diagnosis and Testing

In neonates, identifying clinical features allows the selection of individuals for confirmatory genetic testing. A rapid blood test, using fluorescence in situ hybridization, provides evidence of the diagnosis within a few days, followed by full karyotyping within 1 to 2 weeks. Infants with a new or prenatal diagnosis of DS should be examined for signs of CHD and ideally undergo echocardiography.^11,35,36^ In regions where neonatal echocardiography is not easily accessible, screening with a combination of physical examination, ECG, and chest radiography to select infants for further investigation may increase the sensitivity of the initial clinical assessment.^37^

Infants with DS have multiple medical issues, including lower birthweight and smaller head circumference,^38^ that place them at higher risk of mortality, with 7.5% dying in the neonatal period.^39^ Other mortality predictors include certain forms of CHD (eg, pulmonary vein stenosis, Ebstein anomaly, left-sided obstructive lesions) as well as certain associated diagnoses (eg, congenital diaphragmatic hernia). In some studies, the presence of CHD was not associated with a higher in-hospital mortality.^38^ Neonates with DS more frequently require mechanical ventilation and extracorporeal membrane oxygenation (ECMO; needed in 2.3% of neonates with DS in 1 study of 5737 newborns in 43 centers across the United States).^40^

### Optimal Timing of Repair of CHD in DS and the Risk of Developing PH

The type and timing of surgery or intervention required for individuals with DS and CHD depends on the type of CHD, clinical presentation, and individual circumstances or comorbidities (Table 2). For example, optimal timing and type of repair of ASD, VSD, and PDA depends on the size of the defect and associated comorbidities. Some infants with posttricuspid shunts may require pulmonary artery (PA) banding before complete repair because of extenuating circumstances, such as prematurity. As with AVSD, repair of ToF is usually performed early (within 4 to 6 months of birth), even though children with a well-balanced circulation can be repaired at a later stage. A surgical systemic-to-PA shunt or transcatheter stent placement may be required before complete repair to augment pulmonary blood flow in infants with excessive systemic desaturation and to enhance PA development. Individuals with single ventricle physiology require multiple surgeries over their lifetime. These people have a higher perioperative mortality and morbidity compared with those without DS after Fontan surgery, with an in-hospital mortality of 12.3 versus 1.6% (odds ratio, 8.6 [95% CI, 4.4–17.0]).^41^

**Table 2.**
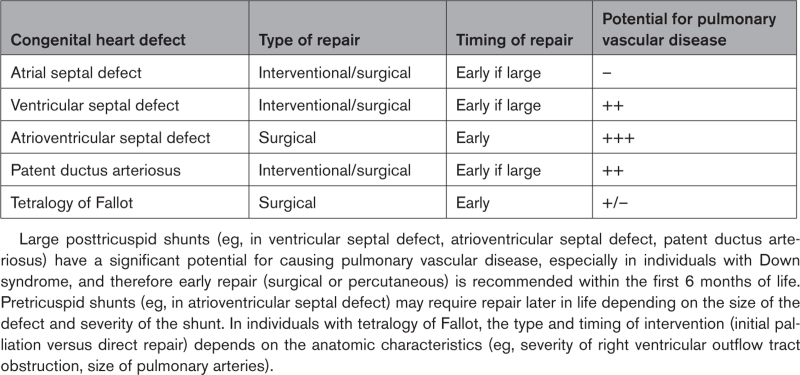
Risk of Pulmonary Vascular Disease and Recommendations Regarding Timing and Type of Repair for Different Forms of Congenital Heart Disease

CHD may be palliated or definitively repaired surgically or percutaneously, assuming reasonable hemodynamics and anatomy. Simple defects, such as ASDs and PDA, may be closed percutaneously when the anatomy is favorable. Balloon valvuloplasty of severely stenotic valve lesions has become the standard of care as an initial procedure. Certain palliative interventions may also be performed percutaneously, including stenting of a ductus arteriosus. Future directions of research include the possibility of transcatheter valve implantation and recently developed percutaneous techniques for the repair of atrioventricular valves.

In general, perioperative morbidity, including length of intubation, intensive care unit admission, and total length of hospital stay, and feeding difficulties are similar for individuals with DS and those without.^42–44^ Mortality is higher in people with DS and single ventricle physiology compared with their non-DS counterparts, however.^41,45,46^ DS is associated with a high incidence of unbalanced AVSD, a diagnosis that may require single ventricle palliation necessitating multiple interventions (catheter driven or surgical) culminating in a Fontan-type operation (in the current era, a total cavopulmonary connection). As with other forms of cardiac surgery, the Fontan operation was initially not considered appropriate for individuals with DS. This is no longer the case, and the presence of a genetic syndrome in itself should not affect the choice of intervention.^47^ In infants with DS and single ventricle physiology, pulmonary vascular protection is essential to achieve a successful Fontan-type repair. Infants with DS are at increased risk of developing pulmonary vascular disease early, which may jeopardize plans to establish a cavopulmonary connection (Glenn/Fontan-type repairs), unless the pulmonary vascular bed is protected from overcirculation with timely PA banding. Careful hemodynamic assessment is imperative in this setting, including invasive assessment in children with a suspicion of PH.

People with DS have a higher lifetime risk of PH. In DS with CHD, especially large posttricuspid (eg, VSD or PDA) or combined pretricuspid and posttricuspid shunts (eg, AVSD), pulmonary arterial hypertension (PAH) commonly develops within the first year of life, with a reported incidence ranging from 6% to 37.5%.^48–51^ The mechanism responsible for the earlier onset of PAH in people with DS and CHD remains unclear and may be related to the genetic syndrome itself and also to common comorbidities (eg, developmental lung disease).

Screening for PH is part of lifelong care in DS (Table 3). PH can be precapillary or postcapillary and its management differs depending on the diagnosis (eg, PAH versus PH related to bronchopulmonary dysplasia versus postcapillary PH in older adults with significant obesity and other metabolic comorbidities or left-sided cardiac lesions). In young children with DS, CHD and persistent PH of the newborn are the most common causes of raised pulmonary pressures,^49^ but with increasing age, DS-associated respiratory problems and left ventricular diastolic dysfunction become more prevalent.

**Table 3.**
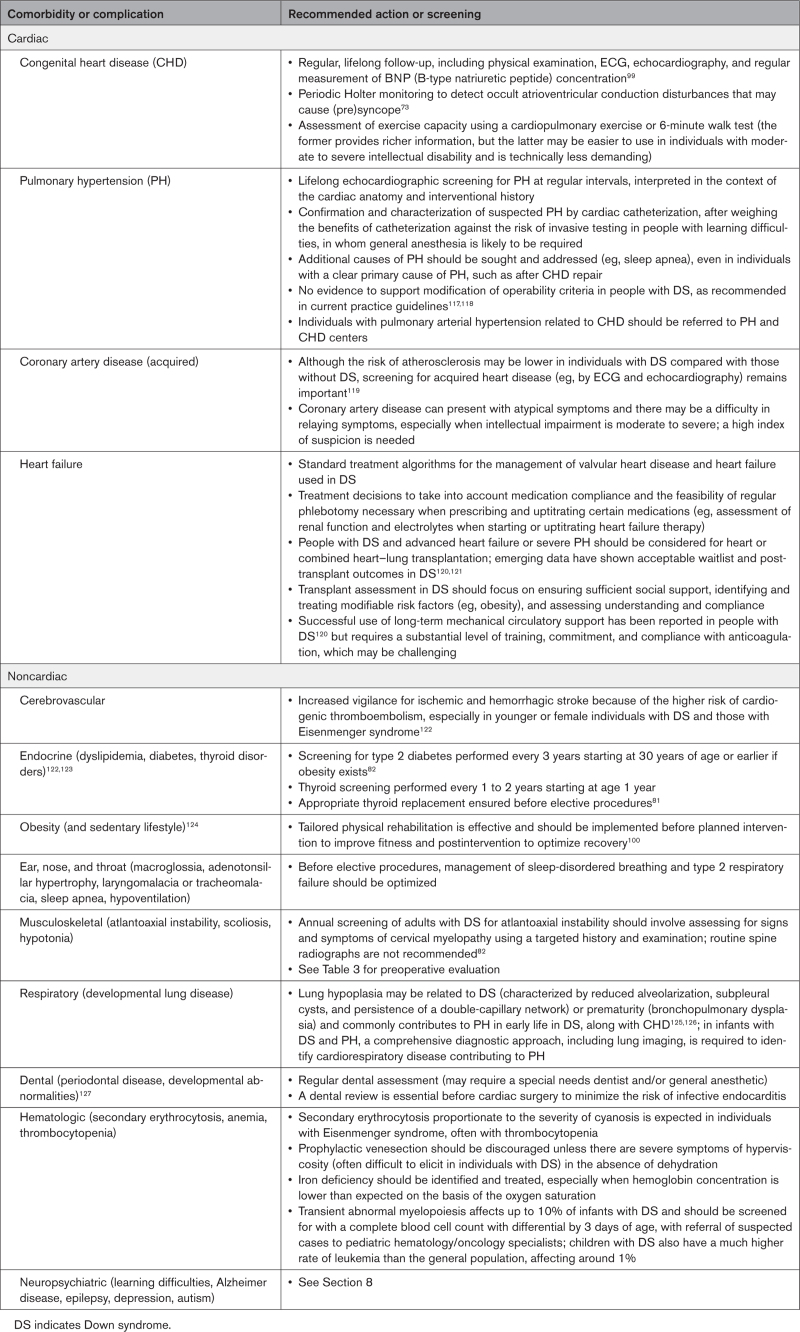
Long-Term Comorbidities and Complications in Adolescents and Adults With DS

In individuals with DS and CHD, early repair is essential in minimizing the risk of developing PAH, which increases perioperative risk and may preclude repair. Established pulmonary vascular disease, and especially Eisenmenger syndrome (a multisystem condition characterized by severe, irreversible pulmonary vascular disease), is associated with high morbidity and mortality and has significant implications in terms of quality of life. Eisenmenger syndrome is most commonly seen in older individuals with DS who may not have benefitted from CHD repair and require specialist care and assessment for PAH therapy. Residual PH after defect repair is also not uncommon and may require therapy in the immediate postoperative period and longer term.^52^

In general, management of pulmonary vascular disease should not differ between those with and without DS.^53^ However, several issues often complicate the care of people with DS and PAH, resulting in a delay in diagnosis and initiation or escalation of treatment. It can be challenging to define symptomatology in individuals with DS. The 6-minute walk test, routinely used to assess exercise capacity and response to therapy in PAH, may be unreliable in individuals with greater levels of intellectual disability and substantial learning disability.^54,55^ There is also a higher prevalence of comorbidities that can contribute to the development and severity of PH and may influence clinical presentation and the response to therapies (eg, obstructive sleep apnea, parenchymal lung disease, hypothyroidism, major depression, obesity, learning difficulties, sedentary lifestyle). Limited data exist regarding the efficacy of PAH therapy in individuals with DS, who were not included in the landmark BREATHE-5 (Bosentan Randomized Trial of Endothelin Antagonist Therapy–5), which established the role of PAH therapies in Eisenmenger syndrome, although cohort studies have since demonstrated the benefit of both endothelin receptor antagonists and phosphodiesterase-5 inhibitors in this population.^56–59^ As a result of these complexities, it is important that people with DS and PAH are identified and referred to specialist centers, where they can benefit from multidisciplinary specialist care and current therapies.

### Perioperative Risks, Complications, and Optimal Care

Individuals with DS may have to undergo 1 or more cardiac or noncardiac surgeries in their lifetime. To manage this population effectively during surgery, detailed preoperative assessment, meticulous procedural care, and management of postprocedural complications and comorbidities are essential.^60^ Echocardiographic studies have shown that even in the absence of overt structural abnormalities, both systolic and diastolic dysfunction are common.^61,62^

The preoperative evaluation of individuals with DS and CHD should consider common comorbidities, including upper airway obstruction, atlantoaxial instability, and endocrine and hematologic complications (Table 4). Difficult airway management and intubation, challenging extubation, and early failure of ventilator weaning have been reported in DS.^63,64^ Where available, the multidisciplinary care of these individuals should include an anesthesiologist with expertise in DS, who can limit anesthesia-related complications in this delicate population. Once preexisting cardiac and noncardiac conditions are identified, careful choice of anesthetic agents should be made. This is determined not only by the pharmacokinetics and pharmacodynamics of the agents but also the unit’s familiarity with these agents. The use of inhalational agents (such as sevoflurane) has been associated with significant bradycardia in DS and when used, require close monitoring and dosing.^65^ Intravenous atropine may be considered if needed. The use of sedatives such as dexmedetomidine to wean individuals from mechanical ventilation after cardiac surgery has been suggested but 1 large study showed no significant influence on mortality, length of stay, or time on the ventilator.^66^ Moreover, bradycardia was more frequent among those receiving sedation.^67^

**Table 4.**
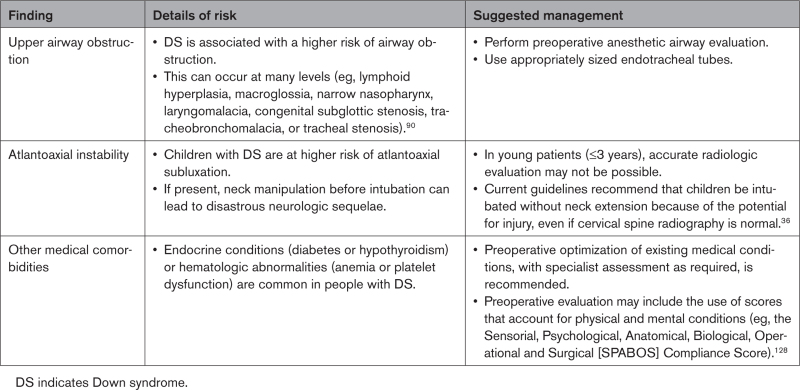
Preoperative Evaluation in DS

Extubation of individuals with DS after surgery should be managed carefully because of the higher rates of postextubation stridor after cardiac surgery. This has been reported in 24% of people with DS in 1 study (additional risk factors included younger age, lower growth percentile for height, and need for reintubation). Subglottic stenosis was also seen in 6.1%.^68^ Opiates, paralytic agents, and sedatives were administered more frequently and for a longer duration in those with DS compared with others.^69^

Contemporary clinical outcomes including mortality after cardiac surgery in children and adults with DS have improved. In fact, people with DS have better survival outcomes than those without DS after many types of cardiac surgery, especially for the well-studied primary repair of complete AVSD.^42–44^ Univentricular repair remains the only exception, where DS is still associated with worse outcome.^41,47^ Although mortality may be low, postoperative complications particular to DS remain substantial and need to be managed well to ensure good outcomes. The prevalence of fever after surgery, for example, is more common in children with DS compared with those without in the first 72 hours. Levels of proinflammatory cytokines (eg, interleukin-6) are often increased.^70^ The risk of nosocomial infection after cardiac surgery is higher in DS, which may be related to immune abnormalities present in DS. Pulmonary infections are particularly common.^71,72^

Whereas supraventricular tachycardia and bradycardia requiring permanent pacing are not uncommon after cardiac surgery (eg, AVSD repair), studies have shown that children and adults with DS do not exhibit a higher risk compared with those without DS.^73^ An exception is perimembranous VSD repair, where those with DS may have a higher rate of permanent pacemaker implantation postprocedure.^74^ Chylothorax is also seen more commonly in children with DS compared with those without DS (16.9 versus 3%), which may be attributable to increased lymphatic permeability. This could also be related to right efferent lymphatic trunk injury; hence, meticulous dissection is needed when operating.^75^ The presence of chylothorax does not appear to substantially affect length of hospital stay or mortality.^76^ The risk of postoperative pericardial effusion is also higher in people with DS. In a large cardiac surgery database on perioperative complications, children with DS had a 25% higher risk of readmission because of pericardial effusion compared with other children.^77^ This was also seen in a study of infants with DS undergoing PA banding.^78^ Anticipating this and performing close monitoring with echocardiography may mitigate preventable poor outcomes.

### Long-Term Complications and Outcomes of CHD in DS

The long-term sequelae of CHD in individuals with DS depend on the underlying lesion and timing of repair. During the last part of the 20th century, there was a dramatic shift, with increasing use of early surgical repair as had already been the standard of care for children without a genetic syndrome. AVSDs comprise a large subset of CHD in DS. Those with transitional (partial) defects (eg, a large primum ASD with atrioventricular valve involvement), as well as those with balanced, complete AVSDs, undergo repair early in life, with excellent short- and long-term results. A sizable minority of people may require late reoperation for residual or progressive left atrioventricular valve regurgitation or stenosis or left ventricular outflow tract obstruction. Whereas the absolute risk of severe adverse outcomes, such as death, may not be higher in those with DS beyond what is accounted for by additional comorbidities (eg, a difficult airway or obstructive sleep apnea), specific consideration is warranted on a case-by-case basis regarding postoperative ventilatory and behavioral management.^71^ Whereas individuals with DS should not be treated differently from others, the indication for surgical intervention should incorporate information on life expectancy and quality of life.^79^ Decisions regarding management can be difficult and are ideally made after best-interest meetings involving an expert multidisciplinary team, those who know the person with DS well, and to the fullest extent possible, the patient.

Providing optimal care for this population requires a willingness to engage in honest and open-minded conversations about medical, psychosocial, and ethical questions. In an earlier era, there was hesitation to perform surgery or other interventions on children with DS. This was partly related to a higher risk because of comorbidities, but also because of a more paternalistic approach to determining what would be best for the individual.^80^ This controversy has persisted into the 21st century, even at leading congenital heart centers.

### Optimal Follow-Up and Long-Term Care for Adolescents and Adults with DS, CHD, or PH

**Figure 1.**
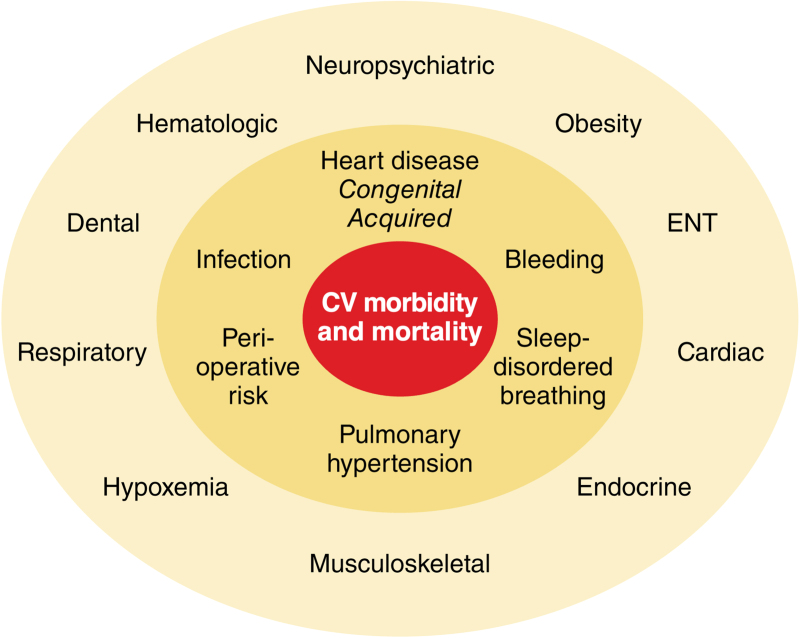
**Cardiac and extracardiac disease in Down syndrome contributing to cardiovascular morbidity and mortality.** CV indicates cardiovascular; and ENT, ear, nose, and throat.

Despite being the most common genetic syndrome, relatively few health care providers outside of tertiary pediatric centers are knowledgeable and experienced in the care of children with DS, who often have complex unmet health needs (Figure 1 and Table 5).^81^ As children with DS and CHD reach adolescence, the process of transition to adult care should be initiated. A structured transition spans several years, ideally from the age of 12 years. The patient should be at the center of the transition process, which should be adapted to their knowledge and intellectual abilities, maximizing their ability to manage their health and promoting independence and social engagement. This process should focus on educating children and their families on both DS and CHD, promoting a healthy lifestyle, and minimizing detrimental behaviors (eg, encouraging regular physical activity and dental hygiene). An important component of the transition process is the structured and safe transfer of care to adult services, which usually happens between 16 and 18 years of age, but should be individualized. This ensures uninterrupted specialist follow-up and effective handover of clinical information, management plans, and contacts of other specialists involved in the person’s care, including clinical geneticists, pediatric and adult congenital cardiologists, dentists, psychiatrists, otolaryngologists, gastroenterologists, neurologists, and rehabilitation specialists.^82^

**Table 5.**
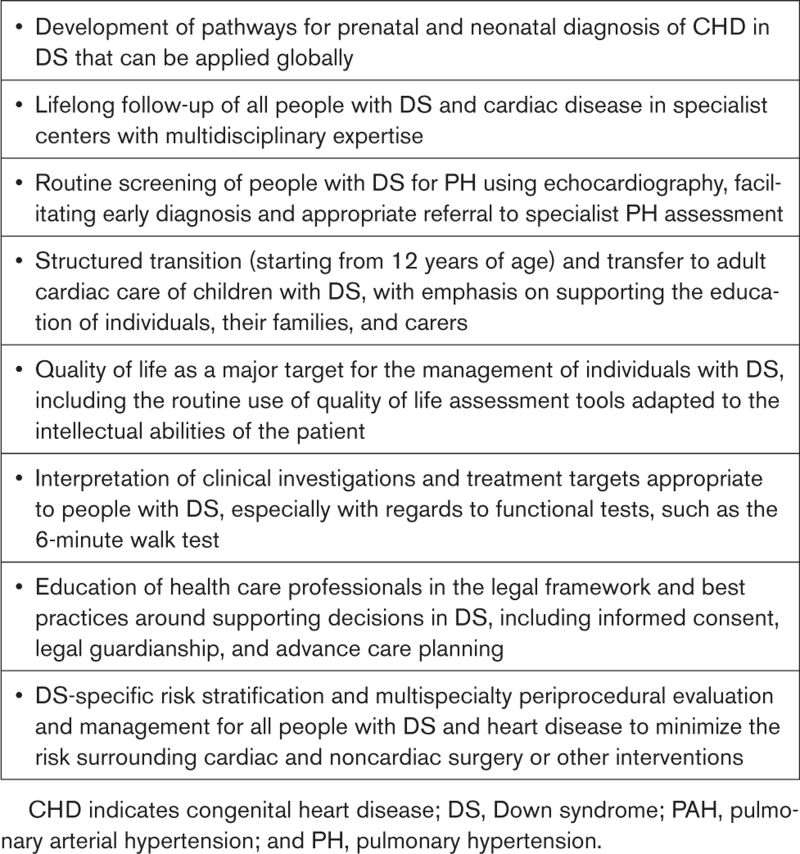
Unmet Clinical Needs of People With Down Syndrome

Individuals with DS and CHD may develop cardiac lesions in adulthood that may benefit from reintervention. Surgical reintervention in adults with DS and CHD can carry an increased risk of complications, especially in the presence of comorbidities including obesity, severe sleep apnea, or PH. Adults with DS and CHD should be followed in expert centers, where they can undergo careful evaluation of perioperative risk.^83^

**Figure 2.**
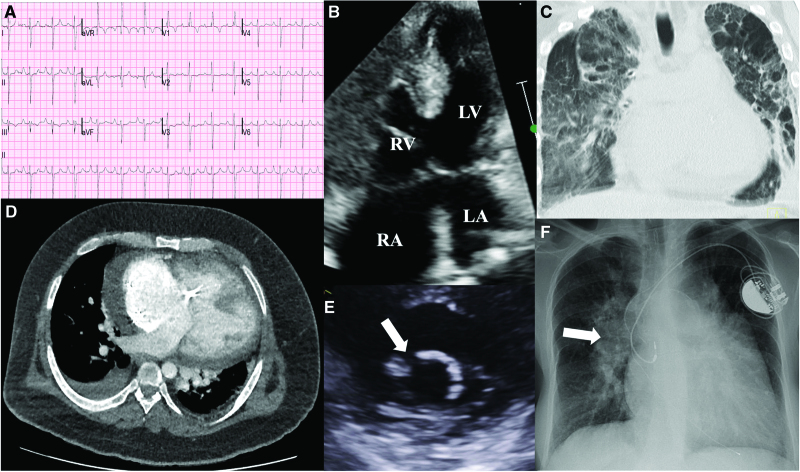
**Manifestations of congenital heart disease in Down syndrome. A**, ECG of an individual with Down syndrome, atrioventricular septal defect (AVSD), and Eisenmenger syndrome. There is right bundle branch block, peaked P waves (P pulmonale), and extreme QRS axis. **B**, A complete AVSD is shown with low velocity bidirectional shunting at atrial and ventricular levels. **C**, Computed tomography scan of the thorax (coronal section) in a person with Eisenmenger ventricular septal defect, displaying gross cardiomegaly along with severe bronchopulmonary dysplasia. **D**, Axial computed tomography image from an individual with Down syndrome, obesity, and Eisenmenger syndrome with complete AVSD and a permanent pacemaker. **E**, Parasternal short-axis view of a trileaflet left atrioventricular valve after AVSD repair. The arrow shows the gap between the 2 bridging leaflets, which is commonly the site of regurgitation. **F**, Chest radiography shows a dual chamber permanent pacemaker in an individual with Down syndrome and Eisenmenger AVSD. There is severe dilation of the pulmonary vasculature, most visible on the right (arrow), and severe cardiomegaly. LA indicates left atrium; LV, left ventricle; RA, right atrium; and RV, right ventricle.

Follow-up investigations for those with DS, CHD, or PH should be performed at regular intervals and can highlight changes in cardiovascular status often difficult to assess solely on the basis of symptoms (Figure 2). Periodic objective assessment of exercise capacity is also recommended in people with DS, CHD, or PH.

Heart failure and heart failure–related hospitalization are more common in people with DS and CHD than in age-matched controls, and heart failure, whether related to congenital or acquired heart disease, is an independent predictor of in-hospital mortality.^84^ Evidence is lacking for conventional heart failure therapies in this population, but standard treatment algorithms are often used, extrapolating existing data from the general population. People with DS and CHD, with or without PH, should be considered for advanced heart failure therapies. In the past, relatively few people with DS and CHD have undergone heart or combined heart–lung transplantation, making this an underutilized therapeutic strategy. The perception that people with DS are not candidates for transplantation may relate to extracardiac comorbidities (including obesity, PH, and an increased risk of infections and acute leukemia); concerns about an individual’s level of intellectual disability, which may affect compliance with follow-up investigations and therapies; or possible bias against people with DS. The presence of DS alone should not serve as an absolute contraindication to transplantation.

### The Influence of Acquired Heart Disease and Noncardiac Comorbidities on the Management and Decision Making Related to Heart Disease in DS

Noncardiac comorbidities in DS (Figure 1 and Table 3) can influence perioperative risk, coping strategies, and the ability to tolerate testing without sedation or anesthesia. Recognition of comorbidities and careful multidisciplinary planning can help reduce periprocedural complications, optimizing clinical outcomes.^83,85,86^ People with DS have a higher risk of prolonged ventilation and length of stay, which often relate to comorbidities such as preexisting respiratory issues.^68,85,87–89^ Altered upper airway anatomy and obstructive sleep apnea with reduced ventilatory drive are highly prevalent, often exacerbated by obesity.^68,90–92^ Obstructive sleep apnea contributes to left ventricular diastolic dysfunction.^61^ Regular screening for cardiovascular disease and risk factor management are important components of management in DS, especially because atypical presentations are common and many individuals have difficulty in relaying symptoms. Unlike the preponderance of hematologic malignancy in DS, the risk of solid organ tumors, including cardiac tumors, may be lower than in other individuals.^93^ Nonetheless, cardiac papillary fibroelastoma has been reported rarely and may require operative management.^94^

### The Influence of Learning Disabilities on the Practical Management of Individuals With DS and CHD

The presence and degree of learning difficulties, pervasive in DS, influences multiple dimensions of health care. These include the reporting of symptoms and presentation of complications, the ability to perform disease surveillance, effective promotion of positive health behaviors (such as engaging in regular physical activity), capacity assessment, and person-centered decision making. Superimposed psychological issues (anxiety in particular), challenging behavior, and early-onset dementia are also common and require considerate planning and staff with adequate training to manage the challenging situations that arise respectfully and effectively.^83,95^ Health care professionals looking after people with CHD and learning difficulties rarely receive formal training and are often not aware of the resources available for these individuals.^96^

The presentation of complications, such as PH and heart failure, in people with DS and CHD may be challenging in the setting of profound learning or communication difficulties. Studies assessing the effects of these issues on timely diagnosis and management are lacking. Specialists often rely on reports from family or caregivers to detect changes in signs or symptoms of disease. Anxiety and challenging behavior can limit physical examination and investigations and require a sensitive approach to assessment. Sedation or anesthesia may be necessary for more detailed investigations, including cardiac magnetic resonance imaging or cardiac catheterization.

Delivering person-centered inpatient and outpatient care is paramount. This involves appropriate training for health care professionals, preparing the clinic environment, minimizing waiting room times, allowing extra time for consultations, providing virtual visits when appropriate, and ensuring that support is in attendance (eg, a family member, a carer, or a sign language interpreter). Other resources can further facilitate the delivery of comprehensive person-centered care, including learning disability specialist nurses.

Adequate follow-up with appropriate serial testing of individuals with DS and CHD is likely to require additional support from the clinical team. Exercise testing may be difficult for many people unable to comply with instructions and alternative modalities (eg, 6-minute walk test versus cardiopulmonary exercise testing may be preferable).^54,55^ In the subset of patients who can complete a cardiopulmonary exercise test, this may provide a reliable assessment of exercise capacity, although age-, sex-, and body size–specific nomograms are not validated and should be used with caution in this cohort.^97^ In particular, the commonly applied normative equations may not accurately reflect the shorter mature stature of adults with DS, with average heights of 157 cm and 145 cm for men and women, respectively.^98^ As stated elsewhere, exercise tests are often submaximal because of factors including poor understanding, limited volition, lack of interest, and orthopedic limitations. Cardiac biomarkers (eg, BNP [B-type natriuretic peptide]) may provide a more objective assessment of cardiovascular status when exercise testing is deemed unreliable.^99^

Regular physical activity should be encouraged in all those with DS to support weight management and to improve cardiovascular fitness and quality of life. Exercise programs for people with DS have been evaluated in small controlled trials and can improve body composition, exercise performance, and autonomic function.^100–102^ Innovative programs that combine physical activity with games may increase the appeal of regular exercise for adults and children with DS.^103^ In addition to exercise programs, broader societal support through education and state-sponsored job opportunities are important to ensure holistic care of people with DS.

Shared decision making should be promoted in individuals with DS and learning difficulties, providing the appropriate support for decision-specific capacity assessment. The delivery of appropriate, informed consent in the setting of wide-ranging learning difficulties can be complex and requires careful, decision-specific assessment by an experienced care provider to avoid ethical or legal pitfalls. DS does not mean the person does not have individual decision making capacity or cannot self-advocate, even in those who are nonverbal. At the same time, one must avoid the scenario where a person who lacks capacity to make a specific decision (eg, whether or not to undergo cardiac surgery) is asked to sign a consent form. If it is determined that a person lacks capacity to make the decision required, it should be established whether there is a surrogate decision maker (eg, a legal guardian). Legally designated surrogate decision makers are not always the same as caregivers but should be very familiar with the individual’s activities of daily life, and ideally share similar cultural, social, and religious values. One should be aware of provider implicit bias (such as projecting personal values or weighing discussions in favor of a certain choice) as well as family implicit bias (including caregiver fatigue or expectations on the basis of historical conversations). The surrogate should be reminded to make the decision that the person would likely make and not the preference of the surrogate. In the absence of a surrogate decision maker, clinician-led best interests decision making should proceed, involving family members, social workers, carers, or others close to the person at the center of care. Ideally, all such individuals should agree on care decisions. In instances where providers and surrogates have different opinions about a decision, despite adequate education and discussion of the issues, the surrogate’s decision should be given priority. It is notable that in many countries, parents of adults do not have any role in serving as a surrogate decision maker unless this has been prespecified. Therefore, in people with substantial learning difficulties, planning around surrogate decision makers should occur before parental responsibility or guardianship ends.

Advance care planning conversations should be initiated early in people with advanced disease by clinicians with adequate resources and training. In line with current legislation and guidance, the presence of a learning disability or DS should never be a reason for setting a ceiling of care (eg, implementation of a do not attempt cardiopulmonary resuscitation order).

### Cardiac Care for Individuals With DS in Low- and Middle-Income Countries

Medical care in low- and middle-income countries (LMICs) varies considerably by region, depending on resource availability and other factors, such as health care policy, education, and medical training. In LMICs where specialist CHD services are available, these are concentrated in large urban centers and not available in rural areas.^104^ Late diagnosis, lack of neonatal intensive care services, and poor access to cardiac surgery play a role in the guarded prognosis of infants with DS and CHD in these settings.^105,106^

The diagnosis of DS in LMICs is mainly on the basis of clinical and phenotypic appearance because prenatal diagnosis and neonatal screening are often limited or unavailable.^107,108^ In 1 African study, only 15% of children with DS who required surgery received appropriate intervention, with delays mainly attributable to low birthweight and late presentation,^109^ as families in LMICs may be unable to identify concerning signs and seek help. Ten percent of children with DS and CHD presented with inoperable disease because of Eisenmenger syndrome,^109^ which in this cohort can be aggravated by the presence of upper airway obstruction (eg, adenotonsillar hypertrophy).

With advances in our knowledge and care of individuals with DS, efforts should be made to ensure that all people with DS worldwide receive timely diagnosis and adequate care. Reference centers should be established to provide support to local teams in ways that would be most appropriate for the local context (eg, telehealth or outreach clinics in remote areas).

Education programs for the population and primary care teams should focus on best practices around pregnancy and delivery and neonatal screening for DS and CHD. Charitable organizations, health care institutions, and governments should be encouraged to provide comprehensive support for families with children with DS (for example, through coordination of peer support groups and facilitated links to specialist services).

Multiple, wide-ranging challenges face LMICs in delivering optimal cardiovascular care. On the macroscopic scale, geopolitical and socioeconomic problems in many LMICs create barriers to health care planning and delivery.^110^ Sociocultural factors and beliefs may contribute to the undertreatment of people with DS and CHD, possibly explaining the higher burden of Eisenmenger syndrome in DS in these areas. There are several barriers to the successful implementation of cardiovascular programs for DS in LMICs, such as lack of infrastructure and specialist care, inadequate health policies and setting of priorities, loss to follow-up, and lack of education of individuals and their families.

Few centers in LMICs undertake care of children with DS, including cardiac surgery.^111^ Strategies to improve cardiovascular and CHD care in LMICs include fostering collaboration through international societies (eg, International Society for Adult Congenital Heart Disease, Asia–Pacific Society for Adult Congenital Heart Disease), fellowship or exchange programs, and charitable hospital links (eg, Children’s HeartLink). This should be combined with resources for training of local cardiologists and CHD specialists. As more children with DS and CHD benefit from surgical and interventional repair, the establishment of transition programs and incorporation of adult CHD into adult cardiology training can ensure specialist care continues into adult life, minimizing loss to follow-up. Treatment for PAH is now affordable in many countries, further prompting screening for early diagnosis and management of PAH. Research should be supported into ways of optimizing care and effectively directing resources in LMICs in a sustainable manner, addressing the complex health needs of people with DS in low-resource settings.^112^

### Future Needs and Challenges in DS Research

This broad review of the literature highlights the limited available evidence on the management of CHD in people with DS, especially in terms of prospective or randomized trials. Individuals with DS and those with learning difficulties in general have often been excluded from randomized trials.^56^ This is often because of issues around consent, which can be overcome by adjusting protocols and training researchers in obtaining informed assent from individuals and their families or carers. Research protocols that include people with DS should provide adjustments, especially when invasive investigations, interventions, or tests that may cause discomfort or require person engagement are planned.^113^

DS-specific normal ranges and variance in outcome measures are required to design and adequately power prospective studies in this group. The choice of outcome measures may also be influenced by the inclusion of people with DS and should account for limitations in self-reporting and confounding from comorbidities (eg, by use of biochemical markers or activity monitors). Moreover, emphasis should be placed on quality of life end points, which are as important an outcome as morbidity and mortality in the DS population. Self-reported quality of life is feasible in some people with DS, although tools specific to DS or frequently associated diseases (eg, CAMPHOR, emPHasis-10 questionnaire) have not been validated in this population and may require adaptation for use.^114^ Few studies have measured quality of life in individuals with DS and cardiovascular diseases.^115^ Instruments measuring quality of life in DS for clinical practice and in research should be developed and validated in this group to support self-reporting in addition to caregiver reports of wellbeing.

International, multicenter registries can be valuable in collecting information and providing pilot data for prospective research. For example, in the MUSES clinical trial, the investigators were able to identify lower use of PAH therapy in people with DS than others with Eisenmenger syndrome and demonstrated the prognostic value of 6-minute walk distance in the overall population (regardless of DS).^116^ Registries for people with DS and PH, including other forms of PAH related to CHD, are urgently needed. Moreover, registries can provide evidence in relation to the optimal follow-up and timing of repair of residual lesions after correction of AVSD and ToF in people with DS.

Improving cardiovascular outcomes in DS also requires specialist centers and funding bodies investing in research focused on comorbidities, including obesity, sleep apnea, autonomic dysregulation, and harmful health behaviors, which can significantly affect long-term cardiovascular health and outcomes in adulthood (Figure 1). A multispecialty, multidisciplinary approach is desirable in the design of such studies enrolling children and adults with DS.

## Conclusions

This review of the literature reflects modern clinical practice, highlighting important advances in several aspects of the care of individuals with DS and CHD or PH over recent years in developed and developing countries. Emphasis is put on education of health care providers and families and structured screening programs for the early identification and management of this distinct population, avoiding pitfalls, accounting for comorbidities, minimizing complications, and optimizing outcome and quality of life of individuals with DS and cardiac disease.

## Article Information

### Acknowledgments

Medical writing support was provided by Eleanor Hobbs of nspm Ltd (Meggen, Switzerland) and Jatta Huotari of eluSCIdate Ltd (Meggen, Switzerland). Eleanor Hobbs assisted A.C. and K.D. on the systematic literature search for the scoping review. The authors thank Andrew Boys and Helen Powell from Down Syndrome International for supporting this work and contributing to establishment of the working group of experts.

### Sources of Funding

Down Syndrome International has received funding from Janssen-Cilag Ltd for the development of an evidence-based cardiac review for people with Down syndrome. Janssen-Cilag Ltd had no influence on the writing of this evidence-based review.

### Disclosures

Dr Dimopoulos has received grants, personal fees, consulting fees, and nonfinancial support from Janssen Pharmaceutical Companies of Johnson & Johnson and grants and personal fees from Pfizer, GlaxoSmithKline, Bayer, and MSD. Dr Constantine has received consulting fees, nonfinancial support, an educational grant, and personal fees from Janssen Pharmaceutical Companies of Johnson & Johnson. Dr Clift has received consulting fees and nonfinancial support from Janssen Pharmaceutical Companies of Johnson & Johnson and personal fees from Bayer. Dr Condliffe has received consulting fees and nonfinancial support from Janssen Pharmaceutical Companies of Johnson & Johnson and personal fees from Bayer and GlaxoSmithKline. Dr Moledina has received consulting fees, honoraria, and nonfinancial support from Janssen Pharmaceutical Companies of Johnson & Johnson and consulting fees from Altavant Sciences. Dr Jansen has received consulting fees and nonfinancial support from Janssen Pharmaceutical Companies of Johnson & Johnson. Dr Veldtman has received consulting fees from Mezzion Pharma. Dr Opotowsky has served on an independent data monitoring committee for and has received consulting fees from Janssen Pharmaceutical Companies of Johnson & Johnson. Dr Giannakoulas has received speaker or consulting fees from ELPEN Pharmaceuticals, Galenica, GlaxoSmithKline, and Janssen Pharmaceutical Companies of Johnson & Johnson and MSD. Dr Chicoine receives royalties for books that he coauthored, published by Woodbine House. Dr Majekodunmi has received speaker fees from Medtronic, GE Healthcare, and Sanofi. Dr Budts has received proctor/speaker/consulting fees from Abbott, Occlutech, and Janssen Pharmaceutical Companies of Johnson & Johnson. Dr Coghlan has received consulting and speaker fees from Janssen Pharmaceutical Companies of Johnson & Johnson, Bayer, and Acceleron Pharma; grants from Janssen Pharmaceutical Companies of Johnson & Johnson; and is a trustee for the Down’s Syndrome Association. Drs Inuzuka, Cua, Alonso-Gonzalez, Cordina, Capone, Scott, D’Alto, Gamero, Gu, Limsuwan, and Broberg and E.T. Lik Wui and J. Namuyonga report no conflicts of interest for this work.

### Supplemental Material

Expanded Online Appendix for Additional Methodologic Details

Tables S1–S7

Figure S1

Appendix

References 129–130

## Supplementary Material


