# Supplementary material for: Cardiovascular Complications of Down Syndrome: Scoping Review and Expert Consensus
Source: Circulation. 2023 Jan 31;147(5):425–41. doi: 10.1161/CIRCULATIONAHA.122.059706 (PMC9977420; doi:10.1161/CIRCULATIONAHA.122.059706)
Supplement: Supplementary file 1 [file cir-147-425-s001.pdf]

SUPPLEMENTAL MATERIAL

Additional details of methodology

The primary aim of this work was to develop a stakeholder-informed set of statements of good practice for individuals with Down syndrome (DS), to guide healthcare practitioners in low-, middle- and high-income countries and inform future guidelines on cardiac disease in DS.

The methodological process included the following key steps: 1) development of a Working Group (WG) of specialists in DS, congenital heart disease and pulmonary hypertension, ensuring representation from low- and middle-income countries (LMICs); 2) agreement on 10 key questions (KQs) of interest in DS and cardiac disease, with the involvement of key stakeholders from Down Syndrome International (DSi), an international disabled people's organization with a membership of organizations and individuals from 136 countries worldwide, committed to improving the quality of life for people with DS; 3) performance of scoping reviews of the literature in DS and cardiac disease to identify the current available evidence and inform the expert comments; 4) peer review and agreement on the proposed statements of good practice by the WG as well as representatives from DSi.

Composition of the Working Group

This initiative was led by the CHAMPION Steering Committee, a UK-based group of experts in congenital heart disease (CHD) and pulmonary hypertension. Members of the WG were proposed by the CHAMPION steering committee as well as expert representatives from DSi and included specialists spanning 16 countries and 6 continents. The working group was divided into a writing group (K. Dimopoulos, A. Constantine, P. Clift, R. Inuzuka, G. Veldtman, C. Cua , E. Tay, A. Opotowsky, G. Giannakoulas, R. Alonso-Gonzalez, R. Cordina, G. Capone, J. Namuyonga, M. D’Alto) and a reference group (R. Condliffe, S. Moledina, K.

Jansen, C. Scott, F. Gamero, B. Chicoine, H. Gu, A. Limsuwan, T. Majekodunmi., W. Budts , G. Coghlan, C. Broberg).

Development of Key Questions

The CHAMPION Steering Committee, in collaboration with expert representatives from DSi, identified and proposed 10 KQs to guide the scoping review of the literature aimed at cardiac disease in DS. Following initial formulation of KQs, these were further developed through consultation with the WG to prioritize topics within CHD that have the greatest impact on patient care. The KQs were developed in accordance with the population, intervention, comparison, outcome, timing and setting (PICOTS) framework for evidence questions (**Supplemental Tables 1-5**).(129)

Scoping Review Search Strategy and PRISMA Flow Chart

A scoping review was conducted using the search strategy outlined in **Supplemental Table 6** in accordance with PRISMA guidelines (**Supplemental Figure 1**). Additional articles were added to the list of identified studies based on recommendations by specialists from the WG.

Review Process of Expert Comments and Statements of Good Practice

Members of the writing group drafted sections of the expert comments using available evidence from the scoping review and their expertise, and proposed statements of good practice. These statements underwent a process of endorsement and open review by the wider WG (including the reference group) and were modified based on WG feedback, so that each outcome fulfilled the following criteria: 1) Statements were in line with available evidence or, in an absence of evidence, expert consensus; 2) Statements apply to high- as well as LMICs. The final set of statements of good practice were approved by the WG, as well as representatives from DSi.

Supplemental Table 1

PICO(TS) Format for Effective Research Questions.(129)

PICO(TS) Element	Description
**P**atient, Population or Problem	The patient(s) of interest. It covers the condition(s), population or subpopulation, disease severity or stage, comorbidities, and other patient characteristics or demographics.
**I**ntervention or Exposure	The specific treatment or approach being evaluated, including dose frequency, method of administration, etc.
**C**omparison	What the intervention above is being compared to. Alternatives include routine care, placebo, drugs, surgery, and lifestyle changes.
**O**utcome	The result(s) of interest. Outcomes may be short, intermediate, and long-term, and may include specific areas such as quality of life, complications, mortality, and morbidity.
**T**iming (if applicable)	The duration of time that is of interest for the particular patient outcome, benefit, or harm.
**S**etting (if applicable)	The setting or context of interest, e.g., primary, secondary, or tertiary medical care.

Supplemental Table 2

Population of Interest for each Key Question Used for Evidence Review.

Key Question	Patients/population of interest
1	Fetuses and infants with DS.
2	Fetuses and infants with DS.
3	People with DS and CHD of all ages (pediatric and adult).
4	People with DS and CHD of all ages.
5	People with DS and CHD of all ages.
6	Adolescents and adults with DS and CHD.
7	People with DS and CHD of all ages.
8	People with DS and CHD of all ages with learning disabilities.
9	People with DS and cardiac disease of all ages in LMICs.
10	People with DS and cardiac disease of all ages.

Abbreviations: CHD, congenital heart disease; DS, Down syndrome; LMIC, low- and middle-income country.

Supplemental Table 3

Intervention or Factor(s) of Interest for Each Key Question Used for Evidence Review.

Key Question	Intervention or Factor(s) of interest
1	Impact on incidence/prevalence of CHD in DS of:<br><br>- Maternal factors<br>- Rates of termination of pregnancy<br>- Improved fetal screening.<br>Changes over time.
2	Fetal ultrasound.
3	Timing of interventions for CHD.
4	Perioperative risk in people with DS and CHD.
5	Long-term complications of CHD.<br><br>Long-term outcomes of CHD.
6	CHD follow-up, including:<br><br>- The process of transition from pediatric to adult care<br>- Need for multidisciplinary and/or expert care<br>- Screening for PH<br>- Use of PAH therapies.
7	Acquired heart disease, non-cardiac comorbidities.
8	Learning disabilities.
9	LMICs.
10	Specific challenges research.

Abbreviations: CHD, congenital heart disease; DS, Down syndrome; LMIC, low- and middle-income country; PAH, pulmonary arterial hypertension; PH, pulmonary hypertension.

Supplemental Table 4

Comparison Group/Intervention of Interest for Each Key Question.

Key Question	Comparison group of interest
1	NA
2	NA
3	People with CHD of all ages without DS
4	People with CHD of all ages without DS
5	People with CHD of all ages without DS
6	NA
7	People without DS (for acquired heart disease)
8	People with CHD/PH without learning disabilities
9	NA
10	NA

Abbreviations: CHD, congenital heart disease; DS, Down syndrome; NA, not applicable; PH, pulmonary hypertension.

Supplemental Table 5

Outcome(s) of Interest for Each Key Question Used for Evidence Review.

Key Question	Outcome(s)
1	Birth prevalence & incidence of CHD in DS.<br><br>Incidence at second trimester fetal screening.<br><br>Proportion of different types of CHD.
2	Rate of diagnosis of CHD.<br><br>Prenatal counselling.
3	Effect on mortality and short- and long-term morbidity, including:<br><br>- Length of intubation, ICU stay and hospitalization<br>- Establishing feeding<br>- Risk of pulmonary hypertension (preoperative).
4	Choice of anesthetic agents.<br><br>Rate of early post-operative complications, including those specific to DS.
5	Rate of late reoperation, eg in AVSD.<br><br>Long-term risk of pulmonary hypertension.
6	Components of transition for adolescents with DS.<br><br>List of investigations used in the follow-up of individuals with DS.<br><br>Risk of PH in people with DS and CHD.<br><br>Impact of PAH therapies in people with DS.
7	Impact and management of non-cardiac comorbidities of DS on peri-procedural risk.<br><br>Prevalence of atherosclerosis and cardiovascular risk factors compared to the general population.
8	Impact of learning disabilities on:<br><br>- Presentation of disease/complications<br>- Disease surveillance<br>- Promotion of positive health behaviors<br>- Capacity assessment /person-centered decision making.
9	Rate of early (prenatal/neonatal) diagnosis and surgical repair of CHD.<br><br>Rate of presentation with complications of CHD, eg Eisenmenger syndrome.

10	Large prospective or randomized trials in CHD where people with DS have been included.<br>Adjustments to research protocols that include individuals with DS, including tools measuring quality of life.

Abbreviations: AVSD, atrioventricular septal defect; CHD, congenital heart disease; DS, Down syndrome; ICU, intensive care unit; PAH, pulmonary arterial hypertension; PH, pulmonary hypertension.

Supplemental Table 6

Scoping Review Search Strategy.

Database	Search Strategy	Search Dates	Filters
PubMed	("Down syndrome"[Title/Abstract] OR "Down's syndrome"[Title/Abstract] OR "trisomy 21"[Title/Abstract]) AND ("cardiac"[Title/Abstract] OR "cardiovascular"[Title/Abstract] OR "heart"[Title/Abstract]) AND (Down Syndrome[MeSH Terms])	1 Jan 1990 – present (July 2020)	Human studies, English language, article type (for PubMed, as below)
Web of Science	((TI=(Down* syndrome) OR TI=("trisomy 21")) AND TI=(heart OR cardi*) AND LANGUAGE: (English) AND DOCUMENT TYPES: (Article)		
Cochrane library	#1 MeSH descriptor: [Down Syndrome] explode all trees<br>#2 (cardiac):ti,ab,kw OR #2 (cardiovascular):ti,ab,kw OR #2 (heart):ti,ab,kw<br>#1 AND #2		

Supplemental Table 7

Studies Reporting the Incidence of CHD in DS.

	Location	Study period	Population	Total, n	CHD, n (%)	AVSD	ASD	VSD	PDA	TOF	CoA	Other	Longitudinal study
**Tubman et al., 1991**(130)	N. Ireland	1987-1989	LB	81	34 (42)	13	7	5	6			3	0
**Khoury and Erickson, 1992**(14)	USA	1968-1989	LB	518	171 (33)								1
**Källén et al., 1996**(17)	Europe	1976-1993	LB	5,581	1,431 (26)	551	98	400	51	50	9	272	0
**Freeman et al., 1998**(21)	USA	1989-1995	LB	227	100 (44)	45	8	35	7	4		1	0
**Torfs and Christianson, 1998**(8)	USA	1983-1993	LB	2,894	1,620 (56)	496	182	185	99	73	28	NA	0
**Frid et al., 1999**(19)	Sweden	1973-1980	LB	219	104 (48)	43	14	34	2		11		
**Rasmussen et al., 2006**(25)	USA	1979-1998	LB	645	266 (41)								0
**Wahab et al., 2006**(20)	Qatar	2000-2005	LB	146	70 (48)								0
**Cleves et al., 2007**(10)	USA	1992-2002	LB	43,463	15,885 (37)	4,671	7,756	5,820		915	399	715	0
**Halliday et al., 2009**(13)	Australia	1988-1990	LB	236	97 (41)								1
	Australia	1998-2000	LB	165	75 (46)								
**Rankin et al., 2012**(22)	England	1985-2003	LB	702	293 (42)	127	52	93		15	8	7	0
**Zhu et al., 2013**(24)	Denmark	1977-1987	LB	1,277	295 (23)								1
	Denmark	1990-2007	LB	987	594 (60)								
**Morris et al., 2014**(16)	Europe	2000-2010	LB, fetal death	7,044	3,068 (44)	977	1,245	1,018	137	115	68	NA	1
**Kim et al., 2014**(23)	Korea	2005-2006	LB	394	224 (57)	37	120	76	69	10	8	8	0
**Scott et al., 2014**(15)	Jamaica	2007	LB	53	42 (79)	23	2	7	7	2	1	0	0
**Stoll et al., 2015**(4)	France	1979-2008	LB, SB, TOP	728	323 (44)	98	82	72	16	10	16	29	0

**Bergström et al., 2016**(26)	Sweden	1992-2012	LB	2,588	1,387 (54)	582	224	307	70	36	90	78	1
**Jaruratanasirikul et al., 2017**(9)	Thailand	2009-2013	LB	149	64 (43)	13	13	16	14	4		4	0
**Brodwall et al., 2018**(12)	Norway	1994–2009	LB	1,251	724 (58)	254	135	184	60	14	10	67	1
**Chua et al., 2020**(18)	Hong Kong	1995-2004	LB	1,010	235 (23)								0

Abbreviations: ASD, atrial septal defect; AVSD, atrioventricular defect; CHD, congenital heart disease; CoA, coarctation of the aorta; LB, live birth; PDA, patent ductus arteriosus; SB, stillbirth; ToF, tetralogy of Fallot; TOP, termination of pregnancy; VSD, ventricular septal defect.

Supplemental Figure 1

PRISMA flow-chart.

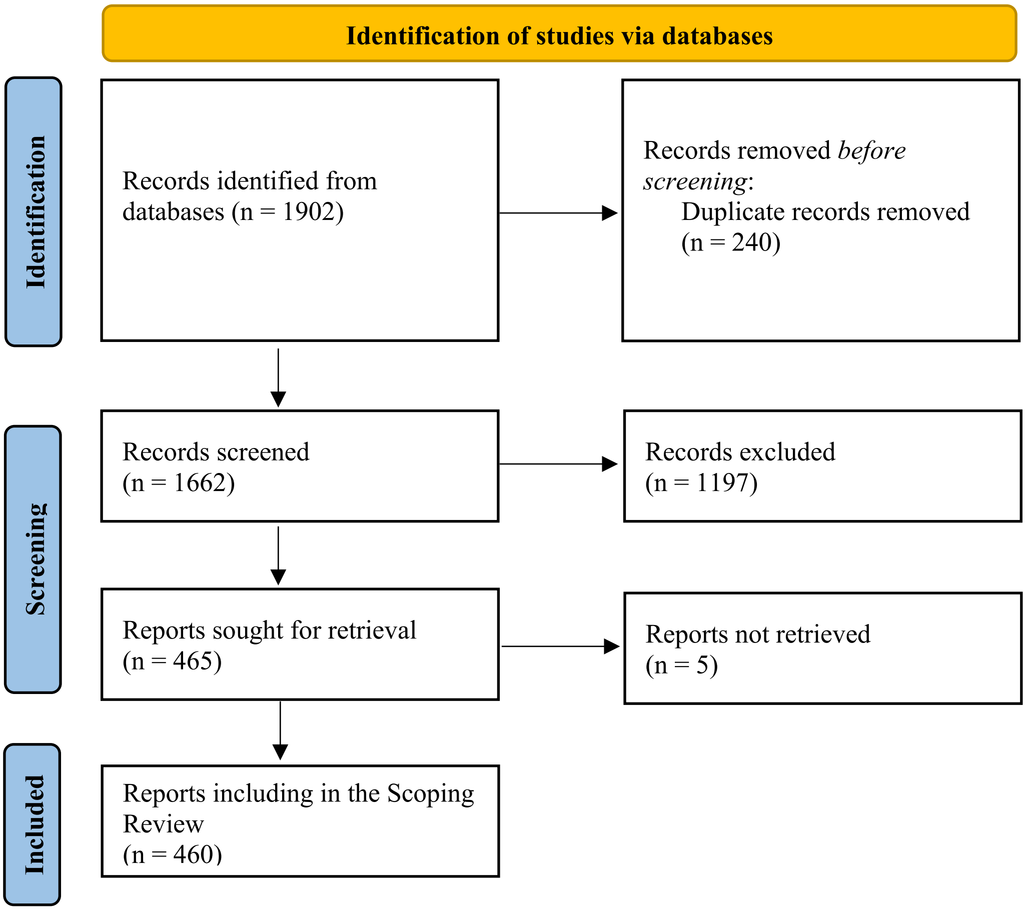

References

Please see main manuscript file for references.

Appendix

Names and affiliations of the CHAMPION Steering Committee:

Konstantinos Dimopoulos MD PhD MSc

Adult Congenital Heart Centre and Centre for Pulmonary Hypertension, Royal Brompton and Harefield Hospitals, and National Heart and Lung Institute, Imperial College London, UK.

Andrew Constantine MBBS MA

Adult Congenital Heart Centre and Centre for Pulmonary Hypertension, Royal Brompton and Harefield Hospitals, and National Heart and Lung Institute, Imperial College London, UK.

Paul Clift MBBS MD BA

Department of Cardiology, Queen Elizabeth Hospital Birmingham, UK.

Robin Condliffe MD

Pulmonary Vascular Disease Unit, Royal Hallamshire Hospital, Sheffield, UK.

Shahin Moledina MBChB

National Paediatric Pulmonary Hypertension Service UK, Great Ormond Street Hospital for Children NHS Foundation Trust and Institute of Cardiovascular Science, University College London, UK.

Katrijn Jansen MD

Adult Congenital and Paediatric Heart Unit, Freeman Hospital Newcastle Upon Tyne Hospitals NHS Foundation Trust and Population Health Sciences Institute, Newcastle University, Newcastle upon Tyne, UK.
